# TRADOC Policy Does Not List Sickle Cell Trait as a Risk Factor for Cold Injury

**Published:** 2024-02-20

**Authors:** Afton D. Seeley, John W. Castellani

**Affiliations:** 1 Thermal Mountain Medicine Division, United States Army Research Institute of Environmental Medicine, Natick, MA

We read with great interest the “Update: Cold Weather Injuries Among the Active and Reserve Components of the U.S. Armed Forces, July 2018-June 2023."^1^ While the update provides valuable insight into the relative number of cold weather injuries incurred by military personnel across the last 5 years, the authors incorrectly stated that the latest 2023 update to Training and Doctrine Command (TRADOC) Regulation^2^ recognizes sickle cell trait (SCT) as a risk factor for cold injury. Current TRADOC policy does suggest SCT screening, driven at least in part by an increased risk of exercise collapse associated with sickle cell trait (ECAST), exertional rhabdomyolysis, and blood clots in austere hot and hypoxic environments.^2^ Yet, no specific policy language exists linking the presence of SCT to the risk or occurrence of cold weather injuries.

SCT is a condition that involves the presence of a mutation on 1 of 2 genes that form red blood cells, while the complementary gene remains unmutated. Because it is typically a benign carrier condition, SCT does not disqualify carriers from military service. Very little data currently exist to support a convincing link between cold weather injuries including hypothermia, freezing injury, or non-freezing cold injury and the presence of SCT. Data from the early 1950s suggests the incidence of frostbite in a small subset of African Americans, who present day tend to disproportionately carry SCT at a rate of 73.1 cases per 1,000 compared to 6.9 in Hispanics and 3.0 in non-Hsipanic Whites,^3^ did not appear greater in those with SCT compared to non-SCT controls.^4^ This very limited sample by no means speaks to a lack of association between SCT and cold injury or cold thermoregulatory adjustments. Undoubtedly there is a profound need to further leverage epidemiological data to improve our understanding of cold injury risk in those with SCT. Additionally, human experimental data is needed to determine if cold thermoregulation in those with SCT uniquely varies from those without SCT, perhaps predisposing them to vascular injury, neurally-mediated cold pain,^5^ or diuresis-induced hypercoagulation.

## AUTHOR AFFILIATIONS

Thermal Mountain Medicine Division, United States Army Research Institute of Environmental Medicine, Natick, MA
