# Supplementary material for: TRADOC Policy Does Not List Sickle Cell Trait as a Risk Factor for Cold Injury
Source: MSMR. 2024 Feb 20;31(2):17. (PMC10959452)
Supplement: Related texts

# In Reply

We thank Dr. Seeley and Dr. Castellani for their interest and careful review of the November 2023 MSMR article “Update: Cold Weather Injuries Among the Active and Reserve Components of the U.S. Armed Forces, July 2018–June 2023.” The editorial team acknowledges the error in interpreting the addition of “sickle cell trait as a risk factor” as an explicit association with cold injuries in the June 2023 update of the TRADOC Regulation on Prevention of Heat and Cold Casualties. We appreciate Dr. Seeley and Dr. Castellani bringing this to our attention. A correction to the original report has been published.
